# Disrupted brain metabolic connectivity in a 6-OHDA-induced mouse model of Parkinson’s disease examined using persistent homology-based analysis

**DOI:** 10.1038/srep33875

**Published:** 2016-09-21

**Authors:** Hyung-Jun Im, Jarang Hahm, Hyejin Kang, Hongyoon Choi, Hyekyoung Lee, Do Won Hwang, E. Edmund Kim, June-Key Chung, Dong Soo Lee

**Affiliations:** 1 Department of Nuclear Medicine, Seoul National University College of Medicine, Seoul, Korea; 2 Department of Molecular Medicine and Biopharmaceutical Sciences, Graduate School of Convergence Science and Technology, and College of Medicine or College of Pharmacy, Seoul National University, Korea.

## Abstract

Movement impairments in Parkinson’s disease (PD) are caused by the degeneration of dopaminergic neurons and the consequent disruption of connectivity in the cortico-striatal-thalamic loop. This study evaluated brain metabolic connectivity in a 6-Hydroxydopamine (6-OHDA)-induced mouse model of PD using ^18^F-fluorodeoxy glucose positron emission tomography (FDG PET). Fourteen PD-model mice and ten control mice were used for the analysis. Voxel-wise t-tests on FDG PET results yielded no significant regional metabolic differences between the PD and control groups. However, the PD group showed lower correlations between the right caudoputamen and the left caudoputamen and right visual cortex. Further network analyses based on the threshold-free persistent homology framework revealed that brain networks were globally disrupted in the PD group, especially between the right auditory cortex and bilateral cortical structures and the left caudoputamen. In conclusion, regional glucose metabolism of PD was preserved, but the metabolic connectivity of the cortico-striatal-thalamic loop was globally impaired in PD.

Parkinson’s disease (PD) is caused by the progressive loss of dopaminergic neurons in the substantia nigra^1^ and the consequent deterioration of the cortico-striatal-thalamic loop^2^. Functional magnetic resonance imaging (fMRI) has been used to image alterations of the cortico-striatal-thalamic loop in PD. Resting state fMRI has shown reduced functional connectivity between cortical and subcortical structures in PD^3^,^4^,^5^. On FDG PET, abnormal metabolic patterns have been observed in connected structures of the striatum, cortex, thalamus and cerebellum^6^. Based on these findings of abnormal concomitant metabolic patterns in these regions, a network abnormality has been suggested, but there has not been a direct demonstration of interregional abnormalities in connectivity. We elucidate disrupted metabolic connectivity among these regions in PD directly using network characterizations and the recently proposed persistent homology framework^7^. Our investigations were first conducted in an animal model because of the higher degrees of homogeneous disease activities compared to human PD.

6-Hydroxydopamine (6-OHDA)-induced neurodegeneration is a well-characterized experimental model for PD. 6-OHDA is selectively taken up by the dopamine transporters of dopaminergic neurons, and it induces retrograde neurodegeneration. Thus, 6-OHDA injections into the striatum or medial forebrain bundle (MFB) can induce nigral degeneration that then results in characteristic behavior abnormalities and immunohistochemical changes^8^. Lundblad *et al*. reported that striatal 6-OHDA injections produced dopaminergic neuronal loss more similar to that seen in PD patients than MFB 6-OHDA injections, as 6-OHDA produces widespread DA denervation extending into the mesocorticolimbic projections^9^. The striatal 6-OHDA model mouse also shows reproducible and characteristic behaviors of side-biased rotation after the injection of amphetamine or apomorphine. Therefore, striatal 6-OHDA-injected mice could be an optimal model of PD for the evaluation of metabolic connectivity.

Quantified regional brain glucose metabolism from FDG PET can represent regional neuronal activity^10^,^11^. Functional metabolic connectivity can thus be investigated by measuring the interregional correlations in FDG uptake and identifying the regions with close correlations using fixed thresholds^12^. However, it is arbitrary to choose a specific fixed threshold, and the single fixed threshold based analysis cannot represent complete interregional correlation. The use of the persistent homology framework and consequent graph filtration provides a new method to investigate multiscale hierarchical networks of interregional correlations among brain areas. Persistent homology assesses network changes over changing thresholds, thus the application of persistent homology-based interpretations to metabolic connectivity data enabled us to identify differences in the connectivity of two groups without a preset threshold^7^. Investigations of brain connectivity using topological interpretations derived from persistent homology have been conducted in human PET and MRI data^7^,^12^,^13^, and we demonstrated that we could successfully distinguish the alterations in brain metabolic connectivity in a pilocarpine-induced rat model of epilepsy^14^. Applications of persistent homology methods to mouse models have not yet been attempted and pose additional challenges because the brain sizes of mice are smaller than those of rats. Herein, we identify alterations in brain metabolic connectivity in PD using a mouse model and apply the same persistent homology-framed network interpretations developed for the interregional correlation of FDG uptake to the 6-OHDA-induced mouse model of PD model.

## Results

### Behavioral analyses and tyrosine hydroxylase (TH) immunohistochemistry

**Figure 1:**
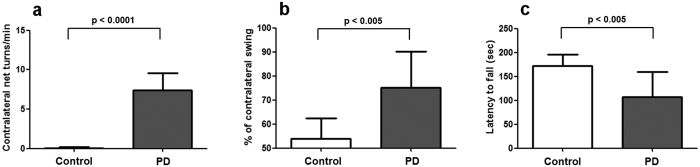
Behavior tests in a 6-OHDA-induced mouse model of PD. (**a**) Apomorphine-induced rotation tests revealed significant side biased rotational movements in the PD group compared with the control group. (**b**) In the elevated body swing test, the PD group showed significantly biased swing movements to the contralateral side of the lesion. (**c**) In the rotarod test, the PD group showed significantly shorter times on the rotating rod than the control group. Error bars indicate standard deviations.

There was no significant weight difference between control and PD groups, either before or 4 weeks after the injection of 6-OHDA or phosphate-buffered saline (PBS) (p > 0.05) (Supplementary Fig. 1). At four weeks after the right striatal injection of 6-OHDA in the PD group or PBS in the control group, the behaviors of all mice were assessed and typical movement impairments were clearly observed in the PD group. All of the mice in the PD group showed significant side-biased rotational movements after apomorphine injection. Contralateral net turns/min in the PD group (n = 14) were significantly higher than those in the control group (n = 10) (mean ± standard deviation, 7.4 ± 2.2 vs. 0.1 ± 0.5, p < 0.0001). In addition, the percentage of contralateral swinging in the PD group was significantly higher than in the control group in the elevated body swing test (EBST) (75.2 ± 14.9 vs. 54 ± 8.3, p < 0.005). Moreover, the latency to fall was significantly shorter in the PD group than in the control group in the rotarod test (106.9 ± 52.9 vs. 172.7 ± 23.1, p < 0.005) (Fig. 1). TH immunohistochemistry showed degeneration of dopaminergic neurons in the right caudoputamen and substantia nigra, further confirmed successful induction of PD (Supplementary Fig. 2).

### Assessment of regional metabolic differences between the PD and control groups

On visual analysis, there was no significant difference between FDG PET images of PD and control group (Supplementary Fig. 3a). Also in the voxel-wise analysis, there were no significant regional metabolic differences between the PD group and the control group, using a false discovery rate (FDR)-corrected p < 0.05. Using a lenient criterion of uncorrected p < 0.001, the PD group tended to display regional hypometabolism in the right caudoputamen (AP:0.3 mm, ML:1.6 mm, DV:2.1 mm, apart from bregma) and right somatosensory cortex (AP:0.9 mm, ML:2.9 mm, DV:2.5 mm, apart from bregma) (Supplementary Fig. 3b).

### Brain regions showing disrupted correlations with lesioning of the right caudoputamen in the PD group

Using an FDR-corrected p < 0.05 for voxel-wise study, there were no voxels showing significant correlations with the right caudoputamen in either group, and there were no voxels showing significant differences in correlation coefficients between the PD and control groups.

**Figure 2:**
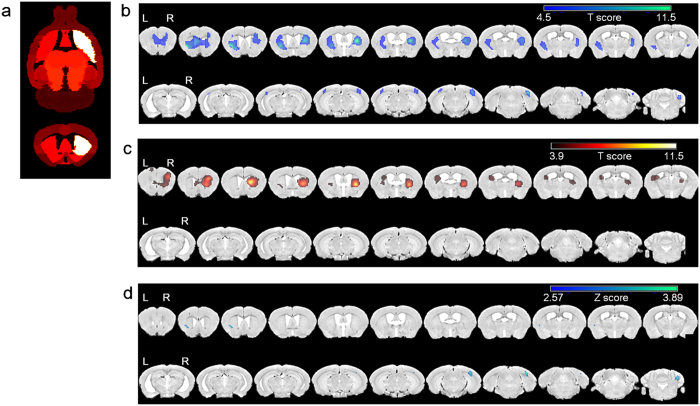
Voxel-wise correlation analyses using the right caudoputamen as a seed area. (**a**) Seed area (right caudoputamen) is shown as the white area on the VOI template. (**b**) In the control group, the both caudoputamen, both motor cortices, both visual cortices and the right cerebellum showed correlations with the right caudoputamen using a lenient criterion of uncorrected p < 0.001 (overlaid image with blue color). (**c**) By contrast, in the PD group, only the both caudoputamen, and both motor cortices showed a tendency toward correlation with the right caudoputamen using the same lenient criterion of uncorrected p < 0.001 (overlaid image with red color). (**d**) Z-score differences over 2.57 (uncorrected p < 0.01) between the two correlation maps from the PD and control groups are displayed in the images. The left caudoputamen, right visual cortex, and right cerebellum showed a tendency toward decreased correlation with the right caudoputamen in the PD group compared with the control group.

However, using a more lenient criterion of uncorrected p < 0.001, in the control group, both caudoputamen, both motor cortices, both visual cortices and the right cerebellum tended to correlate with the seed area and the right caudoputamen. By contrast, in the PD group, only both caudoputamen and both motor cortices tended to correlate with the right caudoputamen (Table 1, Fig. 2a–c). Accordingly, in the PD group, several regions tended to show lower correlation coefficients with the right caudoputamen than the control group, including the left caudoputamen, right visual cortex and right cerebellum (uncorrected p < 0.01, Fig. 2d, Supplementary Fig. 4).

The VOI-based interregional connectivity (r_ij,_ where r_ij_ is a correlation coefficient between i and j, i = right caudoputamen) differences between the PD and control groups were examined. The significance of these differences was determined using the distributions of the differences in correlation coefficients derived from pseudorandom PD/control groups permuted 10,000 times. Based on the permutation test, the PD group showed several areas with lower correlation coefficients with the right caudoputamen than the control group. These areas primarily included the left caudoputamen and the right visual cortex (permuted p < 0.05).

### Persistent brain network homology revealed that the cortico-striatal-thalamic loop was disrupted bilaterally in the PD group

**Figure 3:**
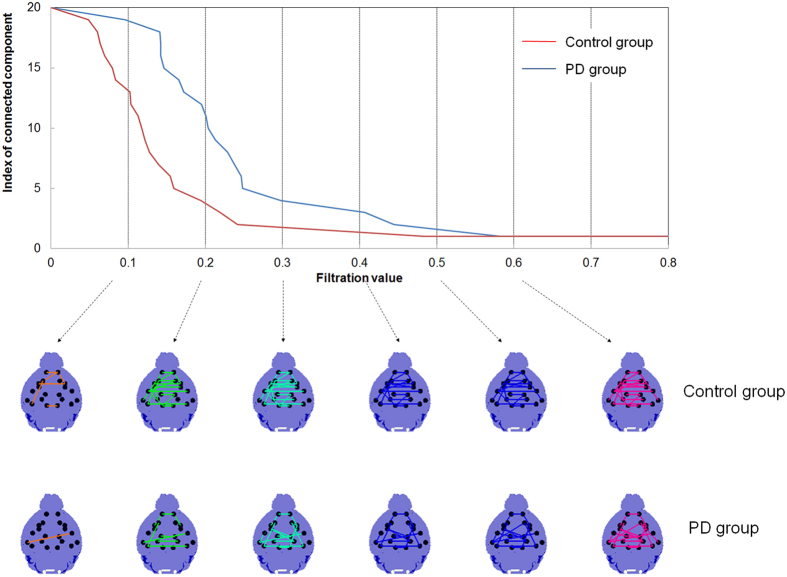
Graph filtration of the PD and control groups. The graphs are shown at six different filtration values, ε = 0.1, 0.2,…, 0.6. Note that areas in the PD group are clustered to a giant single component with larger filtration values (distances) compared with the control group, suggesting globally disrupted and weak connectivity in the PD group.

**Figure 4:**
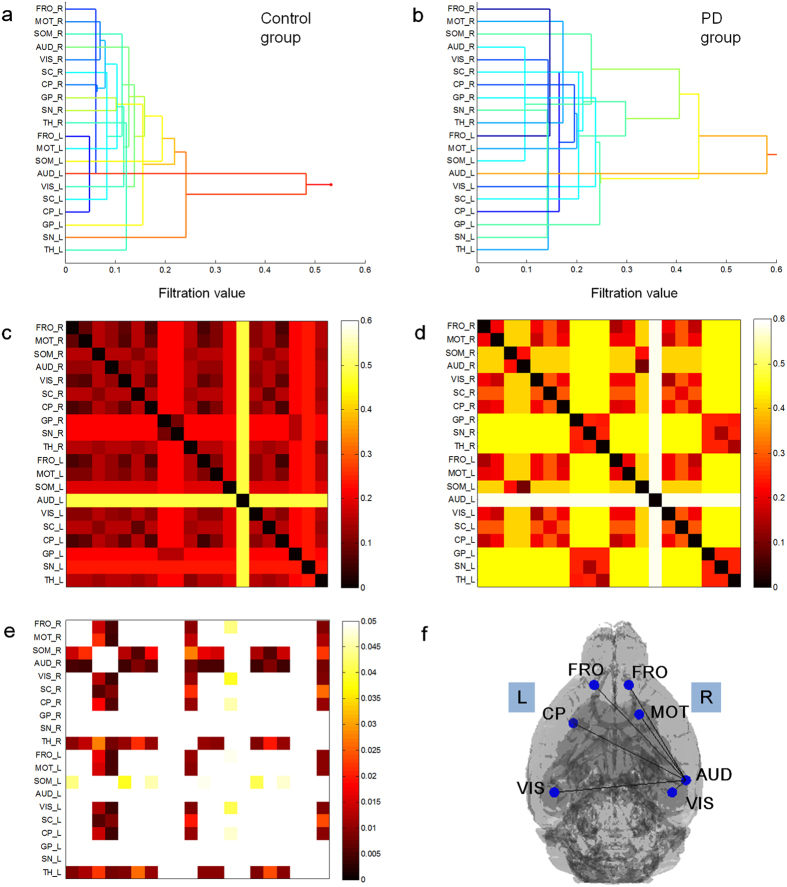
Dendrogram and single linkage distances for the PD and control groups. Dendrograms of the control group (**a**) and PD group (**b**) represent the evolutionary changes in linked areas during graph filtration and distance to be merged into the giant component for each connected component. In the PD group, the areas showed delayed clustering globally. Single linkage matrices as obtained from the distances between the paired areas in the control (**c**) and PD groups (**d**). The scale bar on the right side indicates the single linkage distance. Globally, these single linkage distances (SLDs) were longer in the PD group. (**e**) Using permuted p < 0.05, increased distances between areas in the PD group were found across multiple connections mainly including right somatosensory, right auditory cortex, both caudoputamen and bilateral thalamus. Scale bar on the right side indicates p value. (**f**) Using permuted p < 0.005 based, the most significantly disrupted connections in the PD group were revealed as follows: right auditory cortex-both frontal cortex, right auditory cortex-right motor cortex, right auditory cortex-both visual cortex, and right auditory cortex-left caudoputamen. Abbreviations for areas are described in Supplementary Table 1.

Multiscale networks representing brain metabolic connectivity in the PD and control groups were further compared using the persistent homology framework. Barcodes of the connected components of the networks in both groups were used to visualize network changes during graph filtration using changing filter (distance between pairs of areas) thresholds ε = 0.1, 0.2,…, 0.6 (Fig. 3). The global interregional connectivity among brain regions was looser in the PD group than in the control group across all thresholds (Fig. 3). When single linkage distances (SLDs) were depicted in the form of dendrograms for each group, the PD group showed a slower clustering of areas than the control group (Fig. 4a,b), which also indicated lower connectivity in the PD group.

As with the single linkage matrices (SLMs), SLDs were globally longer in the PD group than in the control group (Fig. 4c,d). Based on our permutation test, connections among areas were looser in the PD group (Fig. 4e, permuted p < 0.05). Representative disrupted metabolic connectivity pairs in PD with the highest differences compared with the control group (permuted p < 0.005, Fig. 4f) include the right auditory cortex-both frontal cortices, the right auditory cortex-right motor cortex, right auditory cortex-both visual cortices, and the right auditory cortex-left caudoputamen.

**Figure 5:**
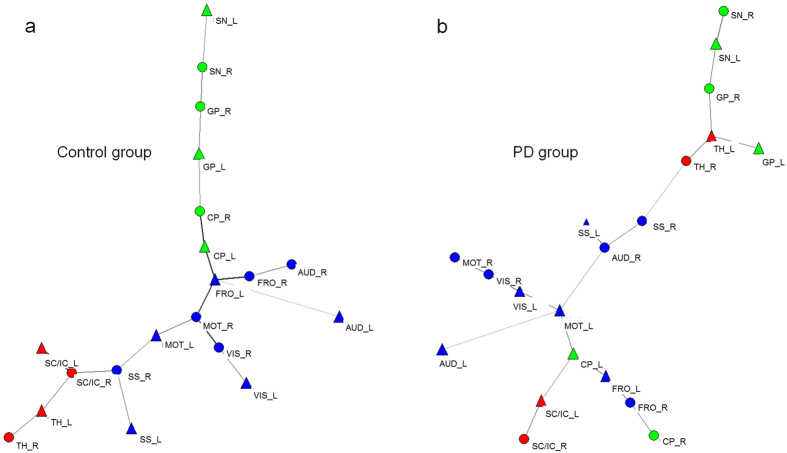
Algorithmic MST graphs of single linkage distance in the control and PD groups. Note that areas of the caudoputamen, globus pallidus and substantia nigra are highly modularized in the control group (**a**) but not in the PD group (**b**) (green: caudoputamen, globus pallidus and substantia nigra, blue: cortical structures, red: the others). Abbreviations for areas are described in Supplementary Table 1.

Finally, algorithmic displays in accordance with the Kamada and Kawai method^15^ of minimum spanning tree (MST) graphs of SLDs showed that in the PD group the caudoputamen, globus pallidus, and substantia nigra were not modularized, whereas they were well modularized in the control group (Fig. 5). Pseudorandom MSTs were also constructed and visualized in this form. The differences between MSTs in the PD and control groups were not random because pseudorandom MSTs from these groups did not yield the specific patterns that we observed here.

## Discussion

In the present study, we demonstrated disrupted metabolic connectivity in the cortico-striatal-thalamic loop in a 6-OHDA-induced mouse model of PD for the first time using FDG PET and metabolic connectivity analyses. Regional metabolic differences were not found between the PD and control groups. However, decreased cortico-striatal correlations in the PD group were demonstrated using VOI-based correlation analyses and permutation testing. Globally disrupted metabolic connectivity was identified in PD using the persistent homology framework.

The utility of FDG PET in PD patients has been evaluated since early the 1990s, but FDG PET is not currently used in routine clinical practice because regional metabolic abnormalities have not been definitively elucidated in PD^16^,^17^. Even in the 6-OHDA-induced rodent model of PD, which displays more uniform characteristics than human patients, regional glucose metabolism has not been consistent, suggesting either decreased metabolism of the ipsilateral primary motor cortex, substantia nigra^18^, or sensory-motor cortex^19^ or unchanged^20^, increased^18^, or decreased^21^,^22^ metabolism in the caudoputamen. In the present study, although there were no significant regional metabolic differences between the PD and control groups after FDR correction, however, using a lenient criterion of uncorrected p < 0.001, the PD group tended to display regional hypometabolism in the right caudoputamen and right somatosensory cortex (Supplementary Fig. 3). Variable metabolic alteration in the caudoputamen could be explained by either the loss of inhibitory nigrostriatal dopaminergic projections and consequent overactivation of the caudoputamen or neurodegeneration in postsynaptic neurons in the caudoputamen after 6-OHDA injection^23^. Considering the heterogeneous results of the referenced studies, including the present study, regarding regional glucose metabolism in both PD patients and rodent models of PD, regional glucose metabolism is unlikely to represent a disease characteristic in PD.

Although regional metabolism in the lesioned caudoputamen was not impaired in the PD group, we found that the correlations between the right caudoputamen and right visual cortex, as well as the contralateral left caudoputamen, were weaker in the PD group. Decreased correlations between the bilateral caudoputamen in the present study were in accordance with recent fMRI studies showing decreased inter-hemispheric connectivity in patients with PD, which is likely associated with asymmetric dopamine depletion in both caudoputamen^24^,^25^. In addition, the decreased connectivity between the visual cortex and the caudoputamen was also in line with previous neuroimaging studies of PD patients. In previous fMRI studies, PD patients showed lower connectivity between the visual cortex and the putamen than control subjects, which plausibly might correspond to observed visual disturbances in PD patients^3^,^4^,^26^.

Encouraged by our findings of areas of lower correlation with the lesioned caudoputamen, we then assessed the global metabolic connectivity of the bilateral cortico-striatal-thalamic loop using the persistent homology framework. Interestingly, we observed that the metabolic connectivity of the cortico-striatal-thalamic loop was widely disrupted bilaterally in the PD group. Among the area pairs showing lower metabolic connectivity, the most significant pairs were between right auditory cortex and several bilateral brain areas, including bilateral frontal, bilateral visual, right motor cortices and left caudoputamen. These areas showed no regional metabolic abnormalities in voxel-wise *t* tests. Impaired connectivity of auditory cortex in PD was reported in the previous connectivity studies, and also widely supported by behavioral evidences. Loose connectivity within the auditory cortex on the lesioned side in the PD group could explain previous observations of contralateral neglect to auditory stimulation in the 6-OHDA-induced rodent model^27^,^28^, and significant reductions in paired-pulse inhibition in the auditory cortex in PD patients^29^. Previous reports have shown beneficial effects of auditory stimulation for motor control in PD patients, also supporting the notion of connectivity impairments between the auditory cortex and motor cortex^30^,^31^. Meanwhile, structures in the left hemisphere, especially the left auditory cortex, showed slower clustering in dendrograms in the control group in the present study. These findings could be explained by the asymmetries of both auditory cortices and the more localized functions of the left auditory cortex. It has been reported that the left hemisphere conducts more intricate patterns of local processing based upon the observations of a lower number of inter connected columnar structures in the left hemisphere^32^. Asymmetries of both auditory cortices are well documented in rodents and humans^33^,^34^,^35^, although the left auditory cortex is considered to show more localized function^34^,^36^.

In the present study, classical subcortical areas comprising the cortico-striatal-thalamic loop, such as the subthalamic nucleus, globus pallidus, pedunculopontine nucleus, and substantia nigra, showed no regional metabolic differences. However, intriguingly, disrupted metabolic connectivity in these motor regulating areas could be identified on MST in the PD group. When MST was displayed in a layout algorithm graph as per Kamada and Kawai, the caudoputamen, globus pallidus and substantia nigra were not modularized in the PD group, whereas those structures were well modularized in the control group.

Formerly, metabolic network abnormalities in PD have been assessed indirectly using PD related metabolic patterns (PDRPs), which are metabolic patterns obtained by principal component analysis in PD patients^37^. PDRPs have been found to show associations with akinetic-rigid disease manifestation and have been validated in multiple independent patient groups^6^,^37^. However, because PDRPs are preset common metabolic patterns in PD patients, their use does not constitute a direct method to demonstrate disruptions in metabolic connectivity. It is thus impossible to evaluate interregional connectivity deficits in PD patients using PDRPs. The persistent homology framework enables the evaluation of such abnormalities in interregional correlations. In the present study, even though there were no regional metabolic differences between the PD and control groups, multiple pair regions with connectivity impairments were revealed. Thus, this method might be used with early-stage PD patients not yet showing the development of specific pattern of regional metabolic abnormalities. Moreover, the method could be utilized in various brain disorders without discrete regional metabolic abnormalities, but with connectivity issues, such as autism spectrum disorder and attention deficit hyperactivity disorder.

The present study is also the first study to map interregional metabolic connectivity of the mouse brain using FDG PET. Recently, interregional metabolic connectivity was successfully analyzed using FDG PET in a rat model of epilepsy^14^. Because the spatial resolution of the PET machine (eXplore VISTA, GE Healthcare, WI) is approximately 1.4 mm^38^, assessing metabolic connectivity using FDG PET is very challenging. However, by using mean values from predefined VOIs which have relatively sufficient volumes (7.24 ± 3.77 mm^3^), metabolic connectivity could be assessed in each group and disrupted metabolic connectivity in the PD group could be revealed in the present study.

The threshold-free persistent homology framework helped to increase our understanding of metabolic connectivity in PD model mice as well as in the control group. As we were interested in differences in SLDs (delta *d*_*ij*_) between the PD and control groups, the distribution of deltas *d*_*ij*_was acquired from permuted pseudorandom PD/control groups (10,000 times) and the observed delta *d*_*ij*_ of our sample (PD/control) was compared with these distributions. Permuted p < 0.05 meant type I errors in our observed deltas *d*_*ij*_ can be observed as false positives. As there has not yet been any method developed to correct for multiple comparisons in this type of connectivity comparison, we used the FDR (and non-correction). Because we chose 20 VOIs, thus resulting in 190 *d*_*ij*_ values, simple Bonferroni correction would have resulted in too high a false-non-discovery rate. When we applied a permuted p < 0.05, many area pairs were found, as in Fig. 4e. For display purposes, we delineate the area pairs (Fig. 4f) which showed differences between the PD and control groups with permuted p < 0.005.

We propose that metabolic connectivity mapped using FDG PET plus our persistent homology based framework can be used to identify alterations or abnormalities in interregional connectivity between groups of small animals and possibly humans. Corrections for multiple comparisons should be established to yield consistent results between investigations. The development of other less conservative methods in addition to the FDR is warranted.

In the present study, degree of dopamine degeneration had not been assessed by a quantitative method such as dopamine transporter PET imaging. Although dopamine dependent connectivity has been demonstrated using resting state fMRI in PD patients^39^, it is worth to investigate the influence of the degree of dopaminergic neuron degeneration to metabolic connectivity using multiple groups with a different degree of degeneration in future studies. The adrenergic neurons were not protected during the induction of PD in mouse using 6-OHDA in the present study. It is possible that adrenergic neuron could have been damaged by 6-OHDA injection. The protection of adrenergic neuron using desipramine may enhance the selectivity of dopaminergic neuron degeneration; however, it may not induce an accurate representation of PD since profound adrenergic degeneration is also accompanied in human PD^40^. Moreover, it has been found that combined noradrenergic neuron degeneration contributes to the development of L-DOPA induced dyskinesia, which is a common side effect in human PD^41^.

In conclusion, we showed globally decreased metabolic connectivity in the cortico-striatal-thalamic loop in a 6-OHDA-induced mouse model of PD at rest using FDG PET and persistent homology analysis. These metabolic connectivity impairments were most significant in area pairs in bilateral frontal/visual cortices and ipsilateral motor and contralateral caudoputamen with the seed area in the right auditory cortex on the lesioned side.

## Materials and Methods

### Induction of the mouse model of PD

All animal experiments were approved by the Institutional Animal Care and Use Committee of the Clinical Research Institute of Seoul National University Hospital and were performed in accordance with guidelines from the institute. The mouse model of PD was induced according to previously described methods^22^. Male C57Bl/6 mice were used for the experiments. Briefly, 6-OHDA was injected into the right striatum [anteroposterior (AP) 0.4 mm, mediolateral (ML) 1.8 mm, dorsoventral (DV) 3.5 mm from bregma] at a rate of 0.5 μL/min using a Hamilton syringe (26 G) and a stereotaxic apparatus (David Kopf, CA, USA). The control group was injected with an equal volume of phosphate-buffered saline (PBS) at the same coordinates. The weight of the mice was measured before and 4 weeks after the injection of 6-OHDA or PBS.

### Behavioral analyses

Apomorphine-induced rotation tests were conducted in mice at 4 weeks after 6-OHDA injection. After injection of apomorphine (subcutaneously, 0.5 mg/kg), the rotational movement of animals was observed for 30 minutes. Ipsilateral and contralateral turns were measured by a rotation-measuring device. The results were expressed as contralateral net turns/min (contralateral net turns = non-lesion side turns – lesion side turns). The elevated body swing test (EBST) was also conducted at 4 weeks in accordance with the procedures in our previous report^42^. Briefly, animals were positioned at the vertical axis, inverted approximately 2 cm from the floor, and the numbers of body swings on each side over 10 degrees were counted from the vertical axis for 1 minute. The results from the EBST were expressed as the percentages of contralaterally biased swings. The rotarod test was conducted at 4 weeks according to previously described methods^43^. Briefly, animals were pre-trained on the rotarod over three sessions spanning two days and 120 seconds each time (5 revolutions per minute (rpm) on the first day, 10 rpm and 15 rpm on the second day morning and afternoon). The final tests (three sessions, each lasting 180 s) were performed on the third day at 15 rpm. Between trials, mice were given at least 2 min of rest to reduce stress and fatigue.

### FDG PET

At 4 weeks after modeling, FDG PET scans were acquired in the 6-OHDA-induced PD and control groups. FDG PET scans were performed and reconstructed as previously described^14^. The mice were fasted overnight before the FDG PET scan. Blood glucose level was not measured. On a dedicated micro PET/CT scanner (eXplore VISTA, GE Healthcare, WI, USA), mice were anesthetized with 2% isoflurane at 1–1.5 L/min oxygen flow for 5–10 min. Mice received an intravenous bolus injection (0.2–0.3 mL/mouse) of ^18^F-FDG (18 MBq/mouse), and each mouse was awake for the 35-min period of FDG uptake. Static emission scans started at 45 min, after FDG injection under anesthesia. Emission scan data were acquired for 20 min with the energy window 400–700 keV. The images were reconstructed using a 3-dimensional ordered-subsets expectation maximum (OSEM) algorithm with random and scatter corrections. Attenuation correction was not performed. The voxel size was 0.3875 × 0.3875 × 0.775 mm.

### Preprocessing

Twenty-four PET images were used for the analysis: 14 PET images from the PD group and 10 images from the control group. Individual PET images were spatially normalized to the FDG mouse brain template^44^ (PMOD 3.4, PMOD group, Zurich, Switzerland) using Statistical Parametric Mapping (SPM8, University College of London, London, UK) and the CT mouse brain template from PMOD, which uses the same coordinates as the mouse brain PET template in PMOD. The MR mouse brain template from the National University of Singapore (NUS) was used as the final experimental template^45^. Using the normalization parameters from the PMOD and NUS templates, we normalized our PET images to the MR mouse brain template from NUS (Supplementary Fig. 5). Before preprocessing, all voxels from PET images were scaled by a factor of 10 in each dimension. All PET images were spatially normalized using nonlinear registration after linear affine transformation. Normalized images were smoothened by a Gaussian filter of 6 mm full width at half maximum. Using predefined volume of interest (VOI) templates from the National University of Singapore (NUS)^45^, which consist of 39 anatomical VOIs in stereotaxic coordinates from Franklin and Paxinos^46^, we obtained the FDG uptake of each VOI. Among the 39 VOIs, we chose 10 areas related to the cortico-striatal-thalamic loop (Supplementary Table 1) and divided each VOI into right and left VOIs according in the mid-sagittal plane to yield final FDG uptakes of 20 VOIs. Mean standardized uptake values (SUV) of the VOIs were measured (Supplementary Table 2). The values for FDG uptake in these VOIs were normalized to the cerebellum.

### Comparisons of regional FDG uptake and inter-regional correlations

Mean SUV of VOIs were compared between the PD and control groups. Voxel-wise *t*-tests were conducted to find the differences in regional FDG uptake between the PD and control groups using SPM8. The counts of each voxel were normalized by the mean count of the cerebellum. The false discovery rate (FDR) corrected p value was used to determine the statistical significance. If the data yielded no significant differences between groups, considering that the FDR correction was conservative, for display purposes and to find trends, an uncorrected p of 0.001 was used with an extent of 50 voxels.

Voxel-wise correlations were analyzed between the right caudoputamen as a seed area and all other brain areas as dependent variables in the PD and control groups (Fig. 2a). Correlation maps for the PD and control groups were transformed to Z scores using Fisher’s transformation. Differences in Z score between the correlation maps from the PD and control group were calculated to find voxels with significant differences. When these analyses did not yield any significant differences, we looked for voxels with a tendency toward differences between the PD and control groups (uncorrected p < 0.01) as a preliminary aid for identifying those connections showing differences between the PD and control groups.

### VOI-based comparisons of the correlation maps of the PD and Control groups

To generate brain networks, areas were represented by their VOIs. We acquired intensity-normalized FDG uptake in the 20 VOIs (Supplementary Table 1) from each subject, calculated the Pearson’s correlation coefficients (r) between each pair of the VOIs using population distributions for each group of mice, and we obtained the correlation matrices (20 × 20) for each PD and control animal. Finally, a weighted undirected network matrix was made, where the strength of each connection between VOIs was simply defined as the correlation coefficients (r_ij_) to yield the correlation matrix.

These correlation matrices for the PD and control groups were transformed to Z scores using Fisher’s transformation. To find the significance of r_ij_, we used permutation testing. Randomly reassigned labels (i.e., PD or control group) were permuted 10,000 times from 24 images containing both groups, and interregional correlation matrices were calculated, followed by Fisher’s transformation. We obtained p-values for Type I error via comparison of the observed delta Z score of the (PD - control) group for each r_ij_ and the Z score distribution from permuted data. Here, we found significantly different interregional correlation coefficients between the PD and control groups (permuted p < 0.05).

### Multiscale graph filtration based upon persistent homology to find network differences

Persistent homology is a new multiscale network analysis framework using graph filtration which can show the evolution of network changes over changing thresholds^7^. This method belongs to the family of topological data analyses of network characterization. In persistent homology, the first feature that quantifies the topological properties of network is the number of connected components, called the zeroth Betti number β0^7^,^14^. If all areas are connected to each other in the network, the network’s β0 is equal to 1. The larger β0 is, the more the areas are not connected. The connection between two areas in a brain network is based on the correlation and is usually determined by threshold. Because there is no standard rule for determining a proper threshold, we generated a sequence of networks at every possible threshold of distance (ε) and estimated β0 of all obtained thresholded networks. This procedure is called graph filtration^14^.

We visualize these changes in β0 during filtration using barcodes and dendrograms. The barcodes of β0 monotonically decrease along the varying thresholds from ‘the number of areas’ to one. If any two areas in the network are connected at a smaller threshold of distance (c_*ij*_), the decrease rate of the barcode is faster. The single linkage dendrogram shows the hierarchical connected components of the network along the varying thresholds. In the single linkage dendrogram, the threshold between two areas when they just belong to the same connected component is the single linkage distance (SLD, *d*_*ij*_)^7^. All pairs of SLDs can be represented in single linkage matrices (SLM).

SLMs and barcodes of β0 were constructed with randomly reassigned labels (i.e., PD or control groups) and permuted 10,000 times as a permutation test of our comparisons of correlation maps in the above. Type I errors were calculated by the comparison of the observed differences in SLDs in the PD/control groups for each connection and the distributions in SLD differences from the permuted data. We used p < 0.05 to find interregional connections in VOI pairs showing significantly different SLDs (*d*_*ij*_) between the PD and control groups, and p < 0.005 to describe and explain the VOI pairs.

We also calculated and compared the minimum spanning tree (MST) of the two groups using SLD. The MST is a subgraph of the graph of connected components tree, which was produced by adopting the shortest edges while not allowing redundant connections between areas during graph filtration^7^. In this study, the MST was represented using a layout algorithm graph by Kamada and Kawai^15^ implemented in the Pajek software package (Pajek32 4.01a). This layout algorithm graph by Kamada and Kawai drew once the SLD between areas and iteratively adjusted the positions of the areas while reducing the total energy of the system to a minimum.

### Tissue preparation and tyrosine hydroxylase (TH) immunohistochemistry

At 4 weeks after 6-OHDA injection, animals were deeply anaesthetized and transcardially perfused with PBS followed by 4% paraformaldehyde (PFA). Brains were fixed with 4% PFA solution in PBS (USB Co., OH, USA) for 24 h at room temperature, and embedded in paraffin. Coronal sections (4 μm) of the region covering the striatum (0.5, −0.5 mm from bregma) and substantia nigra (−3.0 mm from bregma) were dewaxed in xylene, and endogenous peroxidase activity was blocked using 0.5% H_2_O_2_ in methanol for 30 min. Sections were then washed in PBS and blocked with normal goat serum or normal horse serum for 30 min at room temperature. The tissue sections were incubated with a 1:500 dilution of rabbit anti tyrosine-hydroxylase (Millipore Co., MA, USA). Slides were incubated with primary antibodies overnight at 4 °C before being washed in PBS and incubated with biotinylated secondary antibodies (anti-mouse IgG) for 1 h at room temperature. The bound antibodies were visualized using an avidin–biotin–peroxidase complex system (Vectastain ABC Elite Kit, Vector), with 3,39-diaminobenzidine as chromogen.

### Statistical analysis

Mann-Whitney *U*-tests were conducted to compare behavioral scores between PD and control groups using MedCalc software (Belgium). A p < 0.05 was considered statistically significant. Voxel-wise unpaired t-tests and correlation analyses using the right caudoputamen as a seed area were conducted using Statistical Parametric Mapping (SPM8, University College of London, London, UK). A corrected false discovery rate p < 0.05 was considered statistically significant. Differences in VOI-based interregional correlation coefficients and single linkage distances between the PD and control groups were evaluated by permutation test using MATLAB 2014b (Mathworks, MA, USA). A permuted p < 0.05 was considered statistically significant (Supplementary Table 3).

## Additional Information

**How to cite this article**: Im, H.-J. *et al*. Disrupted brain metabolic connectivity in a 6-OHDA-induced mouse model of Parkinson’s disease examined using persistent homology-based analysis. *Sci. Rep.* **6**, 33875; doi: 10.1038/srep33875 (2016).

## Supplementary Material

Supplementary Information

## Figures and Tables

**Table 1:** Regions Showing Tendencies Toward Correlation with the Lesioned Caudoputamen.

Region	Control group					PD group				
	Peak T	Peak P	Coordination (mm from bregma)			Peak T	Peak P	Coordination (mm from bregma)		
			ML	DV	AP			ML	DV	AP
R_CP	14.63	<0.0001	2.14	3.06	−0.08	9.98	<0.0001	2.14	3.62	−0.08
L_CP	12.93	<0.0001	−2.33	4.00	0.70	4.50	0.0004	−2.33	3.44	−0.28
R_MOT	5.97	<0.0001	1.68	2.12	1.29	7.59	<0.0001	1.96	2.31	2.10
L_MOT	5.37	0.0003	−0.56	1.94	2.38	4.75	0.0002	−0.84	1.19	2.08
R_VIS	11.74	<0.0001	2.89	1.75	−4.61					
L_VIS	5.61	0.0002	−2.51	1.19	−4.02					
R_CBL	8.66	<0.0001	2.51	2.31	−6.58					

R: right, L: left, CP: caudoputamen, MOT: motor cortex, VIS: visual cortex, CBL: cerebellum, ML: mediolateral, DV: dorsoventral, AP: anterioposterior.
