# Supplementary material for: Disrupted brain metabolic connectivity in a 6-OHDA-induced mouse model of Parkinson’s disease examined using persistent homology-based analysis
Source: Sci Rep. 2016 Sep 21;6:33875. doi: 10.1038/srep33875 (PMC5030651; doi:10.1038/srep33875)
Supplement: Supplementary Information [file srep33875-s1.doc]

**Supplementary information**

**Disrupted brain metabolic connectivity in a 6-OHDA-induced mouse model of Parkinson’s disease examined using persistent homology-based analysis**

Running title: Disrupted metabolic connectivity in mouse PD model

Hyung-Jun Im^1,2^, Jarang Hahm^1^, Hyejin Kang^1^, Hongyoon Choi^1,2^, Hyekyoung Lee^1^, Do Won Hwang^1,2^, E. Edmund Kim^1,2^, June-Key Chung^1^, Dong Soo Lee^1,2^

^1^Department of Nuclear Medicine, Seoul National University College of Medicine, Seoul, Korea; ^2^Department of Molecular Medicine and Biopharmaceutical Sciences, Graduate School of Convergence Science and Technology, and College of Medicine or College of Pharmacy, Seoul National University

Correspondence to:

Dong Soo Lee, MD, PhD

Department of Nuclear Medicine

Seoul National University Hospital

Seoul 110-744

Korea

Tel: 82-2-2072-2501 Fax: 82-2-2072-7690 E-mail: [dsl@plaza.snu.ac.kr](mailto:dsl@plaza.snu.ac.kr)

**Supplementary Table 1.** Selected Volumes of Interest (VOIs) for Analysis

| Name of VOIs | Abbreviation of VOIs for Analysis |
| --- | --- |
| Frontal cortex | FRO_R, FRO_L |
| Motor cortex | MOT_R, MOT_L |
| Somatosensory cortex | SOM_R, SOM_L |
| Auditory cortex | AUD_R, AUD_L |
| Visual cortex | VIS_R, VIS_L |
| Superior + Inferior colliculus | SC_R, SC_L |
| Caudoputamen | CP_R, CP_L |
| Globus pallidus | GP_R, GP_L |
| Substantia nigra | SN_R, SN_L |
| Thalamus | TH_R, TH_L |

R : right side, L : left side

**Supplementary Table 2.** Mean SUV of VOIs in control and PD groups.

|  | Control | | PD | |
| --- | --- | --- | --- | --- |
| Volume of interest | Mean | SD | Mean | SD |
| FRO_R | 2.30 | 0.21 | 2.35 | 0.38 |
| MOT_R | 2.11 | 0.24 | 2.15 | 0.36 |
| SOM_R | 2.08 | 0.27 | 2.03 | 0.27 |
| AUD_R | 2.01 | 0.20 | 2.02 | 0.32 |
| VIS_R | 1.94 | 0.20 | 2.00 | 0.36 |
| SC_R | 2.00 | 0.29 | 2.05 | 0.33 |
| CP_R | 2.14 | 0.22 | 2.12 | 0.29 |
| GP_R | 1.88 | 0.21 | 1.86 | 0.26 |
| SN_R | 1.62 | 0.25 | 1.57 | 0.24 |
| TH_R | 1.98 | 0.30 | 1.98 | 0.30 |
| FRO_L | 2.32 | 0.19 | 2.38 | 0.36 |
| MOT_L | 2.13 | 0.20 | 2.18 | 0.31 |
| SOM_L | 2.05 | 0.24 | 2.01 | 0.27 |
| AUD_L | 1.97 | 0.18 | 1.94 | 0.26 |
| VIS_L | 1.95 | 0.17 | 1.99 | 0.33 |
| SC_L | 2.00 | 0.30 | 2.02 | 0.34 |
| CP_L | 2.08 | 0.22 | 2.07 | 0.32 |
| GP_L | 1.87 | 0.24 | 1.82 | 0.24 |
| SN_L | 1.59 | 0.23 | 1.52 | 0.20 |
| TH_L | 1.98 | 0.27 | 1.92 | 0.30 |

SD: standard deviation, Abbreviations for VOIs are described in Supplementary Table 1.

**Supplementary Table 3.** Statistical approaches to assess difference between PD and control groups

| Parameters | Statistical analysis | Significant p value |
| --- | --- | --- |
| Behavioral score | Mann-Whitney *U*-test | p < 0.05 |
| Regional metabolism | Voxel-wise unpaired t-test | FDR corrected p < 0.05 |
| Correlation coefficient with caudoputamen | Voxel-wise correlation analysis using right caudoputamen as a seed area | FDR corrected p < 0.05 |
| Correlation coefficient between VOIs | Permutation test | p < 0.05 |
| Single linkage distance between VOIs | Permutation test | p < 0.05 |

VOI = volume of interest, FDG = false discovery rate


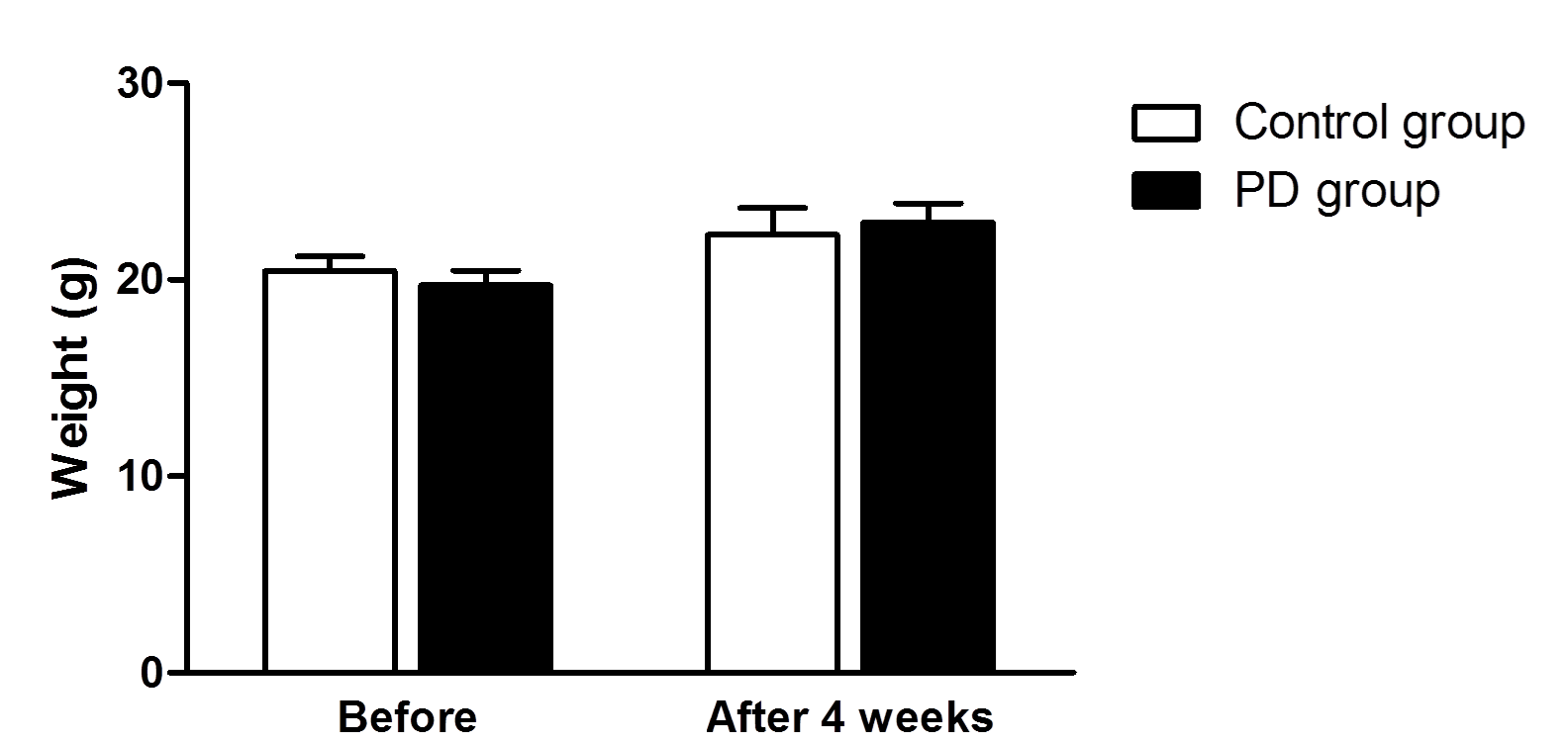


**Supplementary Figure 1.** The weights of the mice were not different between control and PD groups at baseline and 4 weeks after the injection of 6-OHDA or phosphate-buffered saline (PBS)

**
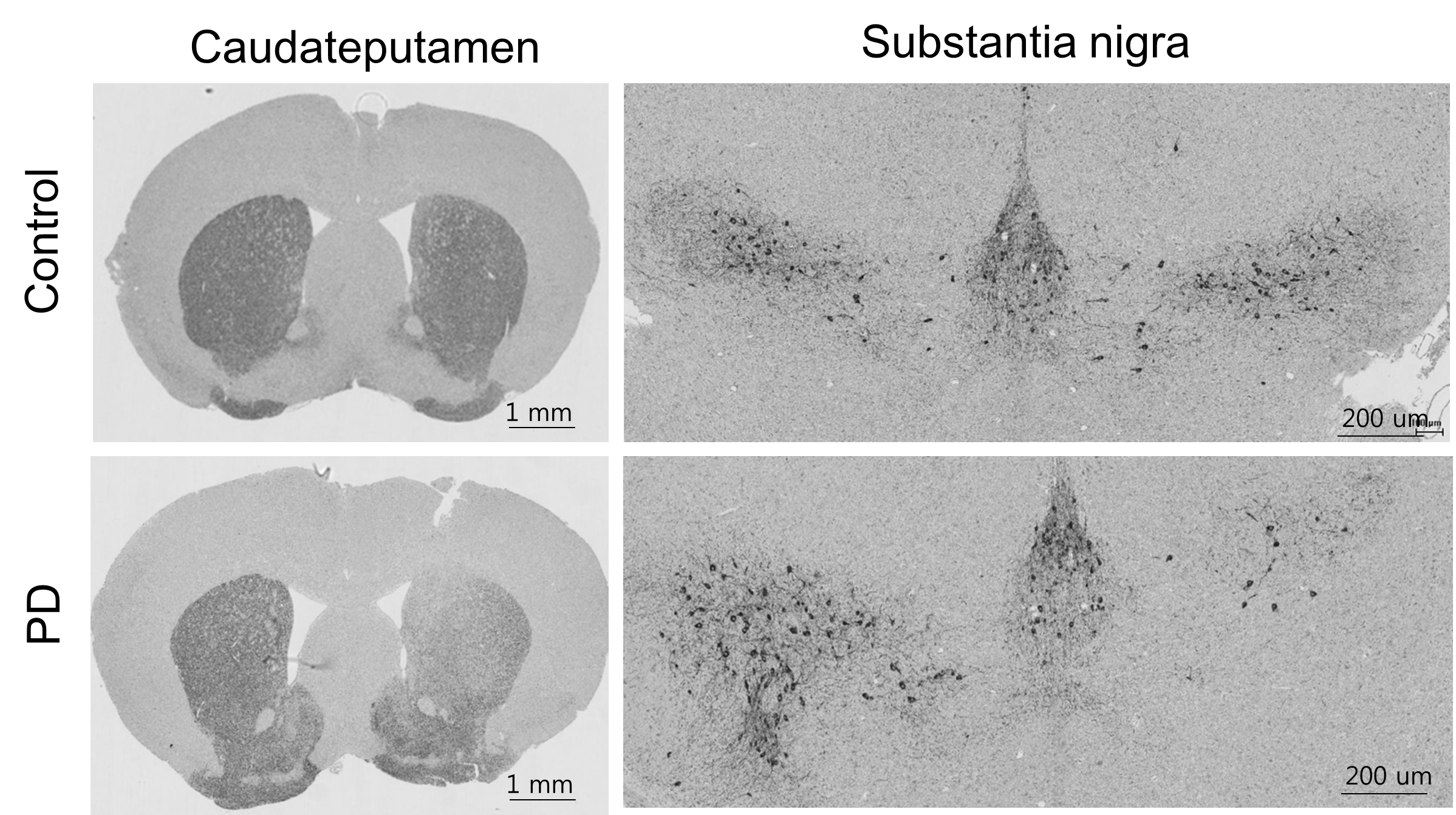
**

**Supplementary Figure 2.**

Four weeks after PBS injection, control group showed symmetric TH stain in both caudoputamen and substantia nigra. On the other hand, 4 weeks after 6-OHDA injection, PD group showed significantly decreased TH immunoreactivity at right caudoputamen and right substantia nigra.


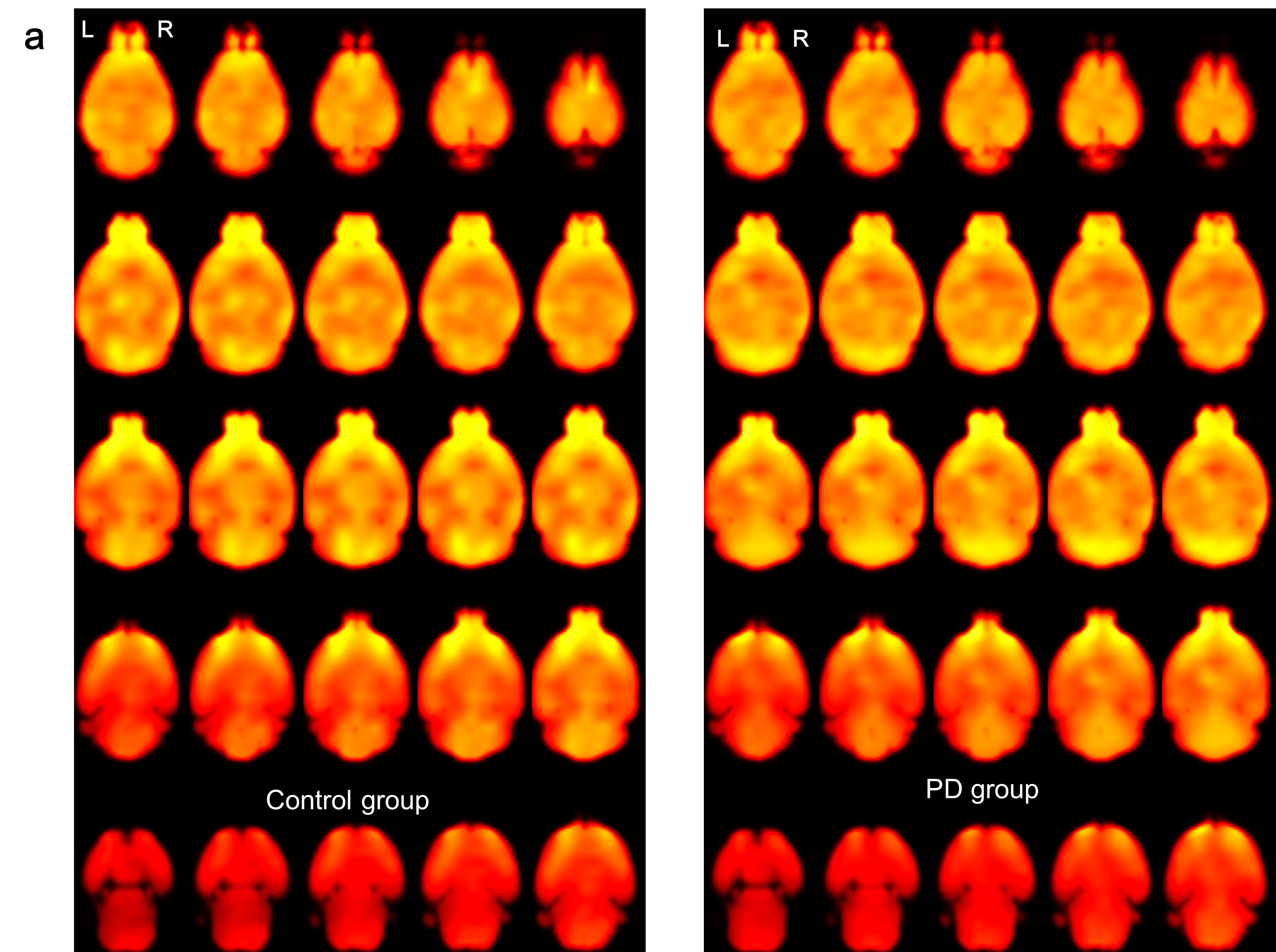


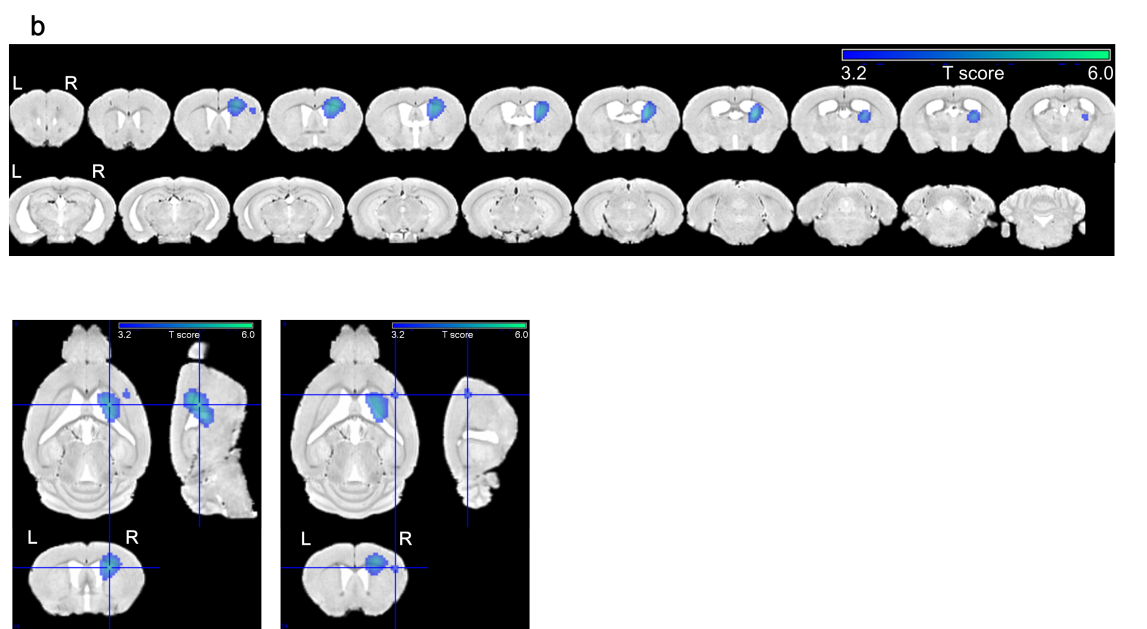


**Supplementary Figure 3.**

(**a**) Representative normalized FDG PET image of control (left) and PD model (right).

(**b**) Voxel-wise t-test between PD and control groups. Using corrected false discovery rate (FDR) criteria (p < 0.05), there was no significant difference between PD and control group. However, using more lenient criteria of uncorrected p < 0.001, there were areas with hypometabolic tendency in PD group which were shown in blue color on MR template. The areas with hypometabolic tendency were in the right dorsomedial caudatepuatamen and right somatosensory cortex (uncorrected p < 0.001). There was no area showing hypermetabolism in PD group compared to control group.


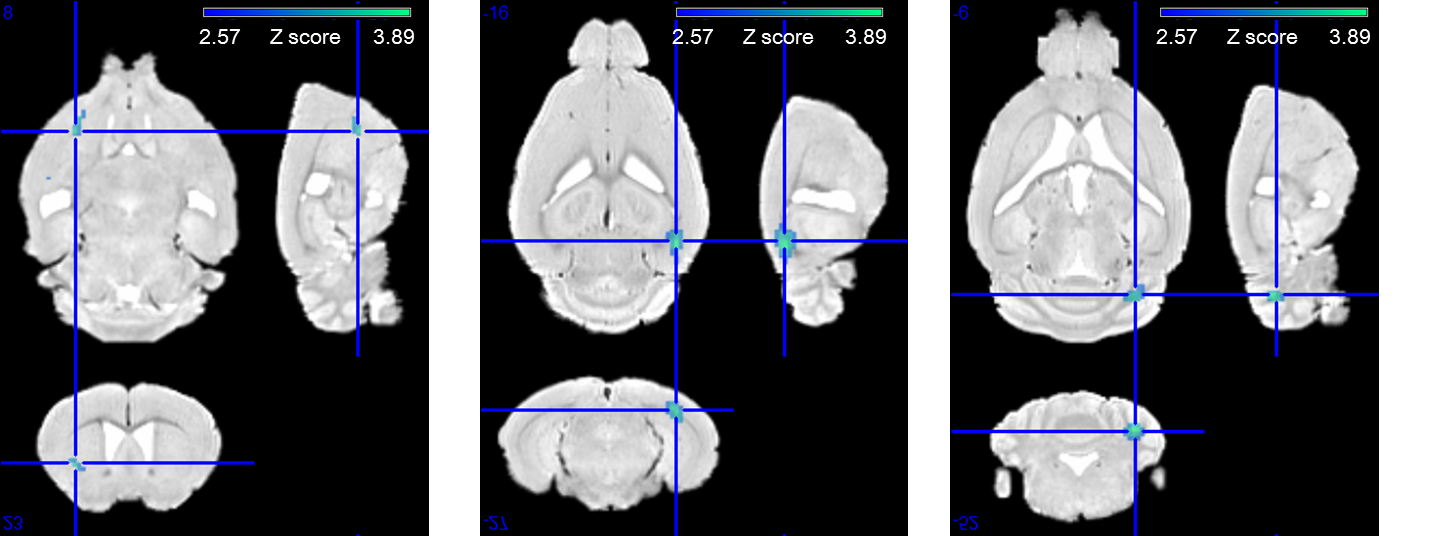


**Supplementary Figure 4.**

Regions having lower correlation coefficient with right caudoputamen in PD group compare to control group; left caudoputamen, right visual cortex, right cerebellum (left to right).


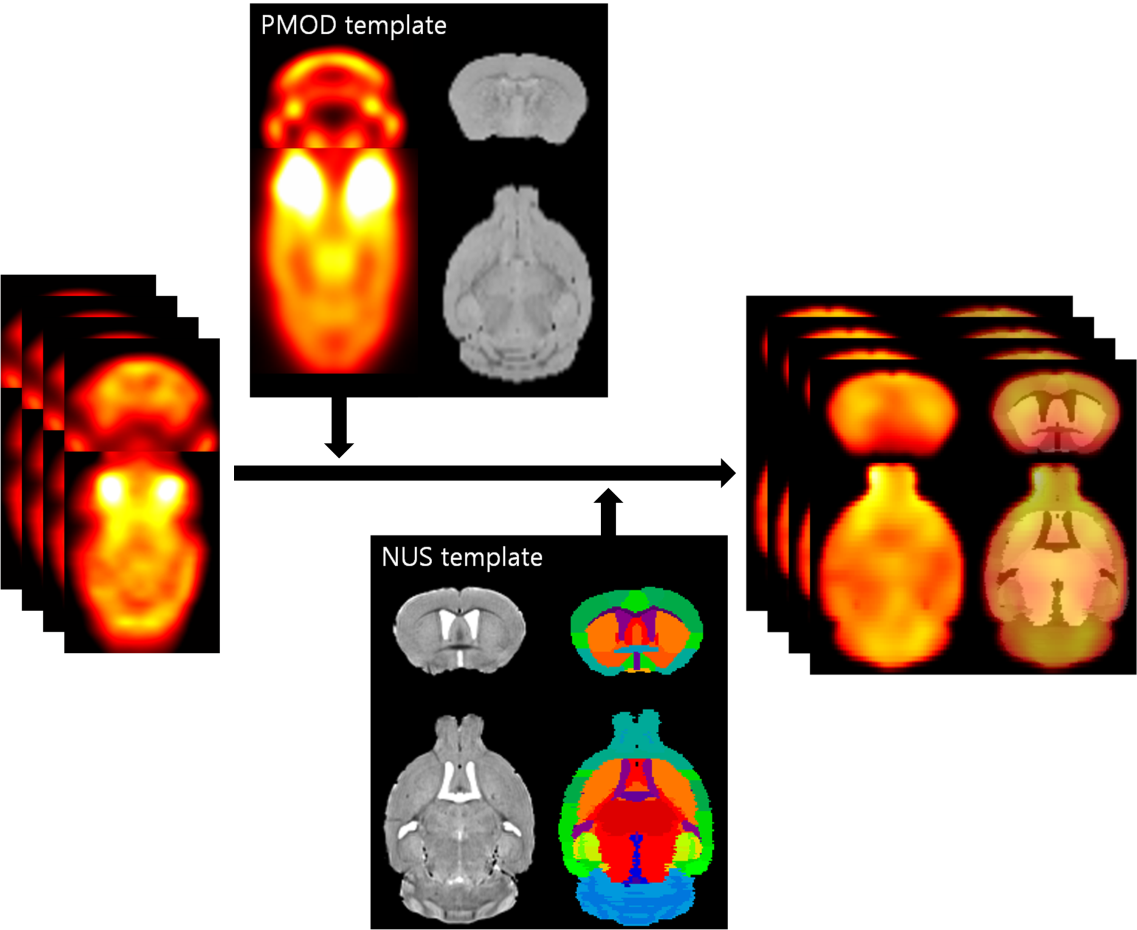


**Supplementary Figure 5.**

Each FDG PET image was spatially normalized to FDG PET template from PMOD. The PET images were normalized again to NUS template. Using predefined VOIs on NUS template, normalized mean count of each VOI from PET images was used for the network analysis.
